# Thailandepsins are new small molecule class I HDAC inhibitors with potent cytotoxic activity in ovarian cancer cells: a preclinical study of epigenetic ovarian cancer therapy

**DOI:** 10.1186/1757-2215-5-12

**Published:** 2012-04-24

**Authors:** Andrew J Wilson, Yi-Qiang Cheng, Dineo Khabele

**Affiliations:** 1 Department of Obstetrics and Gynecology, Division of Gynecologic Oncology, Vanderbilt University Medical Center, 21st Avenue South, B-1100 Medical Center North, Nashville, TN 37232, USA; 2 Department of Biological Sciences, University of Wisconsin-Milwaukee, P.O. Box 413, Milwaukee, WI 53201, USA; 3 Department of Chemistry and Biochemistry, University of Wisconsin-Milwaukee, P.O. Box 413, Milwaukee, WI 53201, USA; 4 Vanderbilt-Ingram Cancer Center, Vanderbilt University School of Medicine, 691 Preston Building, Nashville, TN 37232, USA

**Keywords:** HDAC inhibitors, Thailandepsins, Romidepsin, Ovarian cancer

## Abstract

**Background:**

New treatment strategies are emerging to target DNA damage response pathways in ovarian cancer. Our group has previously shown that the class I biased HDAC inhibitor romidepsin (FK228) induces DNA damage response and has potent cytotoxic effects in ovarian cancer cells. Here, we investigated newly discovered HDAC inhibitors, thailandepsin A (TDP-A) and thailandepsin B (TDP-B), to determine the effects on cell viability, apoptosis and DNA damage response in ovarian cancer cells.

**Methods:**

FK228, TDP-A and TDP-B were tested in five ovarian cancer cell lines. Cellular viability was measured by 3-(4,5-dimethylthiazol-2-yl)-2,5-diphenyltetrazolium bromide (MTT) assays. Immunofluorescence assays were used to assess activated caspase 3. Western blots were performed to detect protein expression of PARP cleavage, pH2AX, P-glycoprotein and tubulin acetylation.

**Results:**

Treatment with TDPs decreased cell viability at nanonomolar concentrations in four of the five ovarian cancer cell lines studied. Similar to FK228, both TDP compounds exerted minimal effects on NCI/ADR-RES ovarian cancer cells. Across the four cell lines sensitive to the TDPs, TDP-B consistently had a greater inhibitory effect than TDP-A on cell viability. TDP-B also had relatively greater effects on promoting cell apoptosis and induction of pH2AX (a mark of DNA damage response), than TDP-A. These antitumor effects of TDP-B were of similar magnitude to those induced by an equal concentration of FK228. Similar to FK228, the nanomolar concentrations of the TDPs had little effect on tubulin acetylation (a mark of class II HDAC6 inhibition).

**Conclusions:**

The new small molecule HDAC inhibitors TDP-A and TDP-B are FK228 analogues that suppress cell viability and induce apoptosis at nanomolar drug concentrations. TDP-B showed the most similarity to the biological activity of FK228 with greater cytotoxic effects than TDP-A in vitro. Our results indicate that FK228-like small molecule class I HDAC-biased HDAC inhibitors have therapeutic potential for ovarian cancer.

## Background

Ovarian cancer is the deadliest gynecologic cancer in the United States [1]. Despite aggressive treatment strategies that involve extensive surgical tumor debulking followed by combination platinum-based chemotherapy, the overall prognosis of ovarian cancer remains poor. More than 50% of high-grade ovarian cancers contain abnormalities in DNA damage repair pathways [2] and are theoretically more sensitive to DNA damaging chemotherapy drugs. Our group has an ongoing interest in an approach of targeting histone deacetylases (HDACs), which are chromatin modifying enzymes known to be associated with DNA damage and repair [3-7].

Based on a screen of a panel of small molecule HDAC inhibitors, we have shown that the depsipeptide romidepsin (FK228) to be the most potent in the majority of ovarian cancer cell lines examined [8]. FK228 induced cytotoxic effects measured by induction of a DNA damage response mark, inhibition of cell proliferation and increased cell death. FK228 was isolated from *Chromobacterium violaceum *no. 968, a rare Gram negative bacterium, and recently approved for the treatment of cutaneous and peripheral T-cell lymphomas [9,10]. The primary mechanism of action of FK228 requires reduction of a characteristic disulfide bond that creates a "warhead" thiol group. The thiol binds to zinc in the catalytic center of both class I and class II HDACs and inhibits HDAC enzymatic activity [11]. However, FK228 binding activity to class I HDACs is considerably more potent than to class II HDACs [11].

Thailandepsin A (TDP-A) and thailandepsin B (TDP-B) are newly reported potent HDAC inhibitors discovered from *Burkholderia thailandensis *E264 by means of genome mining by the Cheng group [12]. Similar to FK228 [11], the TDPs share a conserved bicyclic depsipeptide structure, and require a reduced state for the most potent HDAC binding activity [12]. The goal of this study was to determine the anti-tumor effects of these newly discovered "FK228-like" TDPs in ovarian cancer cell lines. We hypothesized that the unique chemical structure of FK228 and compounds with similar properties such as TDPs leads to strong binding in enzymatic assays to class I HDACs and contributes to potent antitumor activity. Here, we show that TDP-B has greater cytotoxic effects than TDP-A in ovarian cancer cells, but is similar overall to FK228 in its antitumor biological activity.

## Methods

### Compounds

Romidepsin (FK228) was obtained from Gloucester Pharmaceuticals, Celgene Corporation, Cambridge, MA. The TDPs, TDP-A and TDP-B, were discovered, patented, and provided by the Cheng group [12]. Dimethyl sulfoxide (DMSO) (Sigma Chemical Co., St Louis, MO), at a concentration of 0.01%, was used as a vehicle.

### Cell culture

The epithelial ovarian cancer cell lines SKOV-3, OVCAR-8 and NCI/ADR-RES were grown in RPMI 1640 medium supplemented with 10% fetal bovine serum and penicillin/streptomycin, and passaged using standard methods [8,13]. SKOV-3 (American Type Culture Collection, Manassas, VA), OVCAR-8, and NCI/ADR-RES cells (National Cancer Institute, Bethesda, MD) are well-characterized as part of the National Cancer Institute 60 Cancer Panel [14,15]. UWB1.289 (Brca1 null) and UWB1.289 + BRCA1 (Brca1 wild type) cell lines (American Type Culture Collection) were maintained as previously described [16]. All cell lines were used within 6 months of receipt and tested negative for mycoplasma prior to the following experiments.

### Cell viability assays

Cell viability assays using 3-(4,5-dimethylthiazol-2-yl)-2,5-diphenyltetrazolium bromide (MTT) (Sigma) were conducted using established methods [8]. SKOV3 and OVCAR8 cells were seeded at a density of 1250 cells/well in 384 well plates (Corning Life Sciences, Lowell, MA), while NCI/ADR-RES cells were seeded at 1750 cells/well. Absorbance was measured at 540 nm using a Spectramax M5 spectrophotometer (Molecular Devices, Sunnyvale, CA). Cell viability was determined by measuring the percentage of viable treated cells compared to controls.

### Immunofluorescence

SKOV-3 ovarian cancer cells were fixed, permeabilized and stained with anti-caspase 3 (Cell Signaling Technology, Inc, Danvers, MA) as per established protocols [8,17]. Binding of the primary antibody was detected with Alexa Flour anti-mouse IgG 488 secondary antibodies (Invitrogen, Carlsbad, CA), as appropriate. Nuclei were counterstained with DAPI (Millipore Corp., Billerica, MA). Fluorescence microscopy images were captured in 24-bit TIFF format with a Zeiss Axiocam HRC camera (Carl Zeiss MicroImaging Inc.) using Zeiss Axiovision 3.1 software, and analyzed as previously described [8,17].

### Western blot analysis

Whole cell protein isolation, Western Blotting and signal detection were performed as described from NCI/ADR-RES, OVCAR-8 and SKOV-3 ovarian cancer cells [13,18]. For histone extraction, cell pellets were resuspended in an extraction buffer: PBS containing 0.5% Triton X100 (v/v) (Sigma), 2 mM phenylmethylsulfonyl fluoride (Sigma) and 1:100 dilutions of protease and phosphatase inhibitor cocktails (Sigma). Cells were lysed on ice for 10 min and centrifuged for 10 min at 2000 rpm at 4°C. The pellet was resuspended in 0.2 N hydrochloric acid (Sigma) supplemented with protease and phosphatase inhibitors and acid extracted overnight at 4°C. The samples were centrifuged for 10 min at 2000 rpm at 4°C, and the supernatant was collected for further analysis.

The following antibodies were used: anti-phospho H2AX (Ser^139^) and anti-histone H3, (Millipore); anti-P-glycoprotein (PgP), anti-acetylated α-tubulin, anti-α-tubulin and anti-β-Actin (Sigma); and anti-Poly (ADP-ribose) polymerase (PARP) (Cell Signaling, Beverly, MA). Where applicable, corresponding levels of β-Actin, α-tubulin or histone H3 were determined to ensure equal protein loading.

### Statistics

Unless otherwise indicated, values are the mean + SE of at least three independent experiments with p < 0.05 relative to control using the Student's *t *test.

## Results

### Newly discovered small molecule HDAC inhibitors TDP-A and TDP-B are FK228 analogues

**Figure 1:**
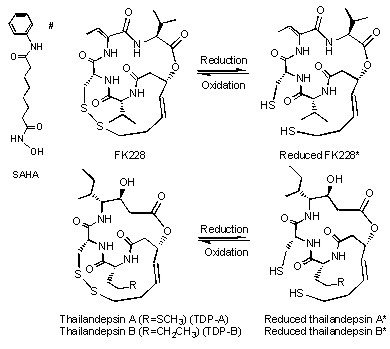
**Chemical structure of HDAC inhibitors**. Thailandepsin A (TDP-A), thailandepsin B (TDP-B) and FK228 are depsipeptides characterized by a bicyclic structure containing a signature disulfide bond; the prodrugs can be activated by cellular reduction, indicated by a star sign (*). SAHA is a hydroxamic acid marked by a pound sign (#)

Our group's experience with FK228 has led us to speculate that there are unique features of FK228 that contribute to its potency in ovarian cancer cells. The strong inhibitory activity against class I HDACs compared to class II HDACs is well-known [11]. It is possible that this preferential class I HDAC binding contributes to the cytotoxic effects of FK228. The TDPs and FK228 share a conserved bicyclic depsipeptide structure that differs significantly from SAHA, a hydroxamic acid (Figure 1). In this study we asked if TDPs, HDAC inhibitors with similar chemical and class I HDAC binding properties, produce similar biological effects in ovarian cancer cells.

### TDP-A and TDP-B inhibit cell viability at nanomolar concentrations

The antiproliferative and cytotoxic activities of TDPs have been compared to FK228 in sulphorhodamine B cell growth assays in the NCI60 panel of cancer cell lines [12]. Of the seven ovarian cancer cell lines present in the panel, SKOV-3 cells were the most sensitive, NCI/ADR-RES cells were profoundly resistant and OVCAR-8 cells exhibited an intermediate response. Therefore, we chose this subset of the NCI60 panel for further investigation of the cytotoxic effects of the TDPs. In addition, we included the well-characterized isogenic Brca1 null and wild type (WT) cell lines in our study, because of the high incidence of BRCA1 dysfunction in ovarian cancer [16].

**Figure 2:**
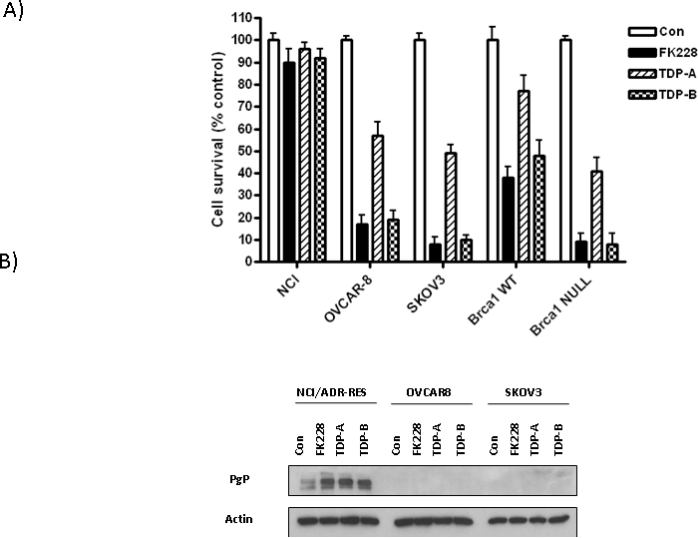
**TDP-A and TDP-B inhibit ovarian cancer cell survival at nanomolar concentrations**. A) Representative graphs of NCI/ADR-RES, OVCAR-8, SKOV-3, BRCA1 wild type and null ovarian cancer cells treated with FK228, TDP-A and TDP-B at a fixed concentration of 10 nM. MTT assays were performed to assess cell proliferation after 72 h of treatment. Each treatment was replicated 6 times. Values are mean + SE for 3 independent experiments. B) Representative Western blot for P-glycoprotein expression in NCI/ADR-RES, OVCAR-8 and SKOV-3 ovarian cancer cells after 24 h of treatment with 10nM FK228, 10nM TDP-A and 10nM TDP-B. 0.1% DMSO was the vehicle control. β-actin was used as a loading control.

First, we performed MTT assays to independently test the effects of FK228 compared to those of the TDPs on ovarian cancer cell viability in vitro. As shown in Figure 2A, TDP-B displayed similar inhibitory effects on cell viability as an equal concentration of FK228 (10 nM) in the three NCI60 cell lines and the isogenic Brca1 wild-type and null cell pair. Although TDP-A (10 nM) induced significant inhibitory effects on cell viability in all cell lines except NCI/ADR-RES, the magnitude of inhibition was consistently less than that observed for TDP-B (Figure 2A). The NCI/ADR-RES ovarian cancer cells were relatively resistant to FK228 and both TDP compounds. Interestingly, we have published that NCI/ADR-RES cells are exquisitely sensitive to SAHA, which is structurally different from the FK228-like compounds [8]. NCI/ADR-RES cell lines highly express the multidrug resistant gene mdr1/ABCB1 that encodes for PgP [15]. FK228 is known to induce PgP [19-21], suggesting that further upregulation of PgP contributes to relative resistance to FK228. Similar to FK228, the TDPs upregulated PgP in NCI/ADR-RES cells (Figure 2B). This activation of PgP may contribute to the NCI/ADR-RES cellular resistance to the TDPs. In contrast, PgP was not detectable in the relatively sensitive cell lines, SKOV-3 and OVCAR-8, either in treated or untreated cells.

Brca1 null cells, which harbor a truncating Brca1 mutation, are known to be more sensitive than their wild-type counterparts to various agents that promote DNA damage [16]. In the MTT assays, the Brca1 status of the cells was similarly observed to determine sensitivity to the small molecule HDAC inhibitors, since FK228 and the TDPs exerted greater effects in the Brca1 null cells.

### TDP-B has a greater effect on the activation of caspase-3 and PARP (markers of apoptosis) than TDP-A

**Figure 3:**
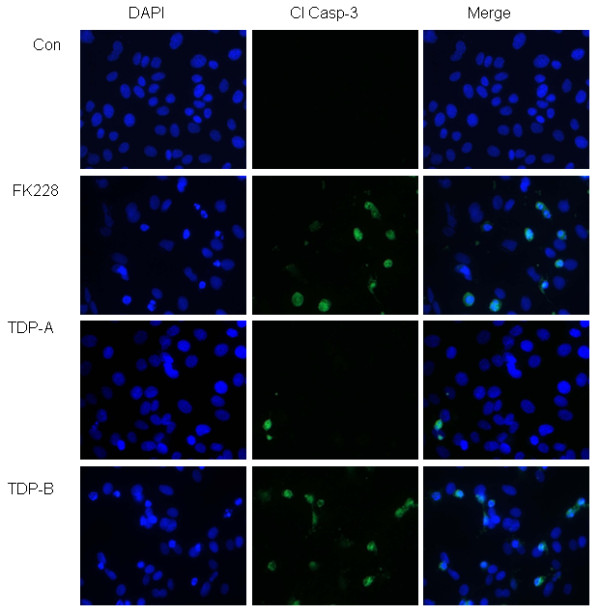
**TDP-B activates cleaved caspase-3 by immunofluorescence**. Representative immunofluorescence staining for cleaved caspase 3 (green) in SKOV-3 ovarian cancer cells treated with 10 nM FK228, TDP-A or TDP-B after 24 h of exposure. The nuclei are stained with DAPI (blue)

**Figure 4:**
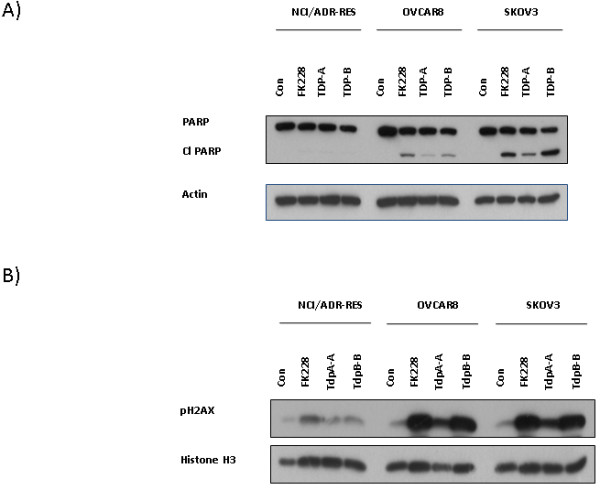
**Representative Western blots show TDP-A and TDP-B upregulate cleaved PARP and pH2AX**. Western blot analysis of A) cleaved PARP and B) pH2AX after 24 h of treatment with 10 nM FK228, TDP-A and TDP-B in NCI/ADR-RES, OVCAR-8 and SKOV-3 ovarian cancer cells. The loading controls in A) and B) were β-actin and histone H3, respectively.

Although TDP-A has previously been shown to exhibit the greatest growth inhibitory effects across cell lines, the cytotoxic activity of TDP-A is lower than FK228 and TDP-B in ovarian cancer cells [12]. One possible mechanism for the observed differences in cytotoxicity is differential induction of apoptosis. Therefore, we examined the effects of TDP-A and TDP-B on apoptosis using two independent assays. Caspase 3 activation measured by immunofluorescence staining for cleaved caspase 3 was similarly induced by FK228 and TDP-B compared to controls in SKOV-3 cells (Figure 3), while fewer TDP-A-treated cells displayed activated caspase-3. Western blot analysis of PARP cleavage was performed to validate these findings. Similarly, cleaved PARP was activated by FK228 and TDP-B to a greater extent than by TDP-A (Figure 4A). As anticipated by the relative resistance to FK228 and the TDPs, there was no evidence of apoptosis induction by these HDAC inhibitors in NCI/ADR-RES cells.

### TDP-A and TDP-B have differential effects on the DNA damage response mark pH2AX

We have demonstrated previously that pH2AX (a sensitive histone mark of DNA damage) is a useful indicator of HDAC cytotoxicity and a potential mechanism for inducing apoptosis in ovarian cancer [8,22]. Prolonged induction of pH2AX foci after genotoxic injury is associated with irreparable DNA damage and apoptosis [22]. Therefore, we evaluated the activation of pH2AX 24 h after exposure to FK228, TDP-A and TDP-B. In relatively sensitive ovarian cancer cells OVCAR-8 and SKOV-3, FK228 and TDP-B induced pronounced upregulation of pH2AX expression compared to controls (Figure 4B). However, in TDP-A treated cells, pH2AX activation was not as robust. In the NCI/ADR-RES cells, pH2AX was minimally activated, reflecting the relative resistance to FK228-like drugs.

### The FK228 analogues TDP-A and TDP-B are class I biased HDAC inhibitors

**Figure 5:**
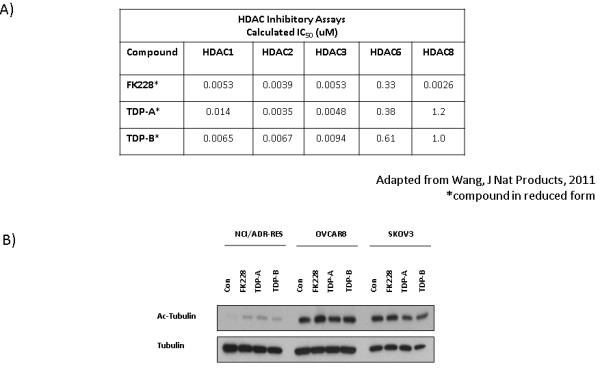
**TDPs are class I biased HDAC inhibitors and do not upregulate acetylated tubulin**. A) HDAC inhibition of HDACs 1, 2, 3 and 6 measured by HDAC enzymatic assays, in which the compounds are reduced in test tubes [12]. B) Western blot analysis of acetylated tubulin in NCI/ADR-RES, OVCAR-8 and SKOV-3 ovarian cancer cells. Tubulin was the loading control.

Inhibition of class I HDACs is known to contribute to DNA damage and cytotoxicity in ovarian cancer cells [7,8]. The cellular responses we observed in the studies presented here support our hypothesis that FK228-like compounds that target class I HDACs exhibit potent antitumor activity. Enzymatic assays comparing TDP-A and TDP-B to FK228 have shown that both TDPs bind to class I HDACs with 30-100 times more potency than to class II HDACs [12] (Figure 5A). Therefore, we tested the effects of TDP-A and TDP-B on a target of class II HDAC6 inhibition, tubulin acetylation [23]. We evaluated tubulin acetylation by Western blot after treatment with TDPs and FK228 at drug concentrations that inhibited cell viability and induced markers of apoptosis and DNA damage response (Figure 5B). However, we found no significant change in acetylated tubulin compared to DMSO treated controls. These results suggest that HDAC6 inhibition is not critical for the antitumor effects of TDP-A and TDP-B, and are in line with previous data showing that low levels of tubulin acetylation do not correlate with the biological activity of HDAC inhibitors in ovarian cancer cells [8].

## Discussion

In this study, we have shown that the recently discovered FK228 analogues TDP-A and TDP-B inhibit cell viability and promote apoptosis in ovarian cancer cells. Furthermore, both agents lead to persistent pH2AX activation, which is a mark of DNA damage-associated cell death [22]. TDP-B is more similar to FK228 in reducing cell proliferation and activating markers of apoptosis and DNA damage, suggesting that TDP-B is more cytotoxic than TDP-A in our system.

Studies such as ours support the underlying hypothesis that FK228 and its analogues have potent antitumor activity due to robust class I HDAC inhibitory function. Interestingly, both the oxidized and reduced forms of TDP-B show potent inhibition of HDAC3/NCOR activity [12]. Previous studies suggest that the class I HDAC3 plays an important role in regulating DNA damage response and is a theoretical target for therapy in cancer cells [7,8,13]. We have shown previously that siRNA knockdown of HDAC3 in ovarian cancer cells contributes to suppression of cell proliferation [13]. The structure of HDAC3 and its unique binding to co-regulator N-COR was characterized recently [24]. One potential mechanism of action of the selectivity of FK228-like compounds is a chemical structure that allows robust binding to HDAC3 and disruption of the HDAC3-NCOR complex. Investigation into 1) precisely how this disruption occurs and 2) whether there is a benefit to selective HDAC3 and other class I HDACs inhibitors is ongoing.

The precise reasons for the differences in activity between the TDPs in our system warrant further study. A recent report of the independently discovered burkholdac B, shown to be identical to TDP-A, had potent picomolar growth inhibitory activity in MCF7 breast cancer cells [25]. These in vitro results of burkholdac B were not consistent with the results of TDP-A published by the Cheng group [12]. Further study of TDP-A is warranted to investigate other mechanisms of growth inhibition such as differentiation and senescence. Future efforts to determine if the observed antitumor effects of TDP-B can be recapitulated *in vivo *will be critical for further development of the TDP compounds.

The efficacy of HDAC inhibitors in cancer therapy is dependent on a number of factors. These factors include: 1) chemical properties of the HDAC inhibitor; 2) strength and selectivity of HDAC inhibition; and 3) phenotypic and molecular features of the cells. Different chemical properties of HDAC inhibitors affect drug availability, pharmacokinetics and pharmacodynamics [3-7]. Furthermore, HDAC inhibitors are known to be more effective when combined with other chemotherapeutic agents [3,4,8]. We have unpublished results showing that FK228 enhances the effects of the DNA damaging agent cisplatin in ovarian cancer cells. Our results are in line with a recent report showing that FK228 is synergistic with DNA damaging agents when given simultaneously [26].

The effects of HDAC inhibitors are not limited to HDAC inhibition. Although the mechanisms are not fully understood, HDAC inhibitors acetylate non-histone molecules that could be exploited for therapeutic purposes. For example, FK228 induces p53 acetylation and may promote degradation of mutant p53 and enhance sensitivity to cytotoxic agents [27]. Since over 90% of high grade serous ovarian cancers harbor p53 mutations [2], FK228 and its analogues may be useful in treating these tumors. Although tubulin acetylation may play a role, we have not shown tubulin acetylation to correlate with the anti-tumor effects of HDACi in this report or in the majority of ovarian cancer cell lines we have screened [8].

Finally, the biological responses to FK228 and other HDAC inhibitors depend on cell type [8,13,28,29]. Malignant cells are more sensitive to HDAC inhibitors than normal cells and hematologic malignancies are more sensitive than solid tumors to single agent treatment with HDAC inhibitors [8,13,28,29]. The NCI/ADR-RES cells are derived from the OVCAR-8 cells [14,15], and represent acquired drug resistance associated with PgP. This cell line pair is a good representation of the clinical status of chemotherapy-resistant ovarian cancers. Thus, determinants of cellular sensitivity and resistance (ex. BRCA mutational status or high PgP expression) as demonstrated here are important considerations prior to treatment.

## Conclusion

The newly discovered class I HDAC inhibitors TDP-A and TDP-B have antiproliferative and proapoptotic biological activity in ovarian cancer cells [12]. TDP-B appears to induce similar cytotoxic effects in vitro as FK228 that are stronger than the in vitro effects demonstrated by TDP-A. The specific components of FK228 and its analogues, along with molecular features that determine cellular vulnerabilities, are critical for the rational development of this chemical class of HDAC inhibitors for the treatment of ovarian cancers and other solid tumors.

## Abbreviations

FK228: Romidepsin; TDP: Thailandepsin; DMSO: Dimethyl sulfoxide; HDAC: Histone deacetylase; pH2AX: Gamma-H2AX phosphorylation; MTT: 3-(4,5-dimethylthiazol-2-yl)-2,5-diphenyltetrazolium bromide; IC_50_: Inhibitory concentration at which 50% of cell viability is reduced; PARP: Poly (ADP-ribose) polymerase; PgP: P-glycoprotein; UWB1.289: Brca1 null; UWB1.289 + BRCA1: Brca1 wild type; WT: Wild type

## Competing interests

DK received an investigator-initiated grant from Celgene Corporation to study FK228 (romidepsin). YQC discovered and holds the patent for thailandepsins (TDP-A and TDP-B).

## Authors' contributions

AJW designed and performed the ovarian cancer cell line experiments, analyzed the data, and helped draft the manuscript. YQC helped conceive the study, provided TDP-A and TDP-B, analyzed the data and helped draft the manuscript. DK conceived the study, oversaw the experiments, analyzed the data, and drafted the manuscript. All the authors have read and approved the final version.
